# Yiqihuoxuejiedu Formula Inhibits Vascular Remodeling by Reducing Proliferation and Secretion of Adventitial Fibroblast after Balloon Injury

**DOI:** 10.1155/2014/849167

**Published:** 2014-05-27

**Authors:** Ming-Jing Zhao, Ai-Ming Wu, Jie Wang, Hong Chang, Yong-Hong Gao, Hui-Min Liu, Xi-Ying Lv, Huan Lei, Qing-Qin Sun, Ying Xu, Ying-Kun He, Shuo-Ren Wang

**Affiliations:** Key Laboratory of Chinese Internal Medicine of Ministry of Education and Dongzhimen Hospital, Beijing University of Chinese Medicine, Beijing 100700, China

## Abstract

Vascular remodeling occurs in atherosclerosis, hypertension, and restenosis after percutaneous coronary intervention. Adventitial remodeling may be a potential therapeutic target. Yiqihuoxuejiedu formula uses therapeutic principles from Chinese medicine to supplement Qi, activate blood circulation, and resolve toxin and it has been shown to inhibit vascular stenosis. To investigate effects and mechanisms of the formula on inhibiting vascular remodeling, especially adventitial remodeling, rats with a balloon injury to their common carotid artery were used and were treated for 7 or 28 days after injury. The adventitial area and **α**-SMA expression increased at 7 days after injury, which indicated activation and proliferation of adventitial fibroblasts. Yiqihuoxuejiedu formula reduced the adventitial areas at 7 days, attenuated the neointima and vessel wall area, stenosis percent, and **α**-SMA expression in the neointima, and reduced collagen content and type I/III collagen ratio in the adventitia at 28 days. Yiqihuoxuejiedu formula had more positive effects than Captopril in reducing intimal proliferation and diminishing stenosis, although Captopril lowered neointimal **α**-SMA expression and reduced the collagen content at 28 days. Yiqihuoxuejiedu formula has inhibitory effects on positive and negative remodeling by reducing adventitial and neointimal proliferation, reducing content, and elevating adventitial compliance.

## 1. Introduction

Vascular remodeling occurs in a variety of diseases such as atherosclerosis, hypertension, and restenosis after PCI. Although these diseases have different causes, they ultimately lead to common pathological changes such as vascular remodeling, which results from and causes pathology in these diseases. Vascular remodeling results from activation, proliferation and migration of cells in the vessel wall, and extracellular matrix synthesis and degradation. Vascular smooth muscle cells (VSMC) in the media have been considered the core of vascular remodeling [1]. However, more and more evidence indicates that “passive” adventitial fibroblasts (AFs) change into “active” myofibroblasts (MFs), which contribute to the arterial remodeling process [2]. Adventitial fibroblasts acquire a migratory phenotype and populate the damaged tissue after stretch injury resulting from a balloon angioplasty [3]. These myofibroblasts produce a thickened and rigid adventitia rich in collagen fibers and further result in a reduction of the vessel lumen size. Thus, adventitia remodeling plays a more important role than previously thought in atheroma formation and restenosis [4]. Inhibiting activation, proliferation, migration, and secretion of adventitial fibroblasts is a new target for the prevention and treatment of vascular remodeling [5].

MF was first discovered in wound healing through electron microscopy. The evidence indicates that MF plays a pivotal role in tissue repair and remodeling and is also a key player in pathological conditions such as hypertrophic scars and organ fibrosis. Professor Jiazhen Liao, a famous cardiovascular disease expert on integrated Chinese and western medicine from China's first generation, gets his inspiration from the surgical wound repair and thinks that the process of vascular remodeling after balloon injury, including thrombosis, inflammatory cell infiltration, and local tissue excessive repair that eventually leads to restenosis, is similar to the pathology of surgical wound repair. He used the wound repair idea and Chinese medicine therapeutic principles of supplementing Qi, activating blood circulation, and detoxifying to develop Yiqihuoxuejiedu formula. The formula is composed of Radix Astragali, Radix Salviae Miltiorrhizae, Flos Lonicerae, Cortex Moutan, and others. Clinical and basic researches, conducted for over 10 years, indicate that the Yiqihuoxuejiedu formula significantly inhibits vascular hyperplasia, lowers blood lipids [6], and reduces neointimal collagen content [7]. In addition, the formula serum promotes VSMC secretion of nitric oxide (NO) and/or reduces the decomposition of NO [8] and inhibits VSMC proliferation and lipid peroxidation injury. A clinical study finds that the formula significantly reduced the probability and cumulative risk of overall cardiovascular events in patients with coronary artery disease (CHD) who underwent stent implantation. The beneficial effect presents at about six months after PCI [9].

According to 2011's PCI guideline [10], secondary prevention after PCI includes antihypertensive therapy with *β* receptor blockers and angiotensin converting enzyme inhibitors (ACEI), lipid-lowering therapy with statins, and antiplatelet/anticoagulant therapy with aspirin and clopidogrel. Of these drugs, ACEI, one of the most important drugs that inhibit cardiovascular remodeling, diminishes the development of atherosclerotic lesions and restenosis after angioplasty through suppressing the generation of angiotensin II (Ang II), which promotes cellular migration, proliferation, and hypertrophy [11]. In this study, ACEI serves as a positive control. Although western medicine therapies have obtained some effects, risks remain and new drugs that prevent vascular remodeling are required. Yiqihuoxuejiedu formula is associated with surgical wound and reduces neointimal hyperplasia after balloon injury. Thus, we hypothesize that Yiqihuoxuejiedu formula inhibits vascular remodeling by reducing adventitial fibroblasts function.

## 2. Materials and Methods 

### 2.1. Animals

Normal male Sprague-Dawley (SD) rats were purchased from the Institute of Laboratory Animal Science, Chinese Academy of Medical Sciences, Beijing, China. SD rats were raised in a specific pathogen free environment at a room temperature of 22°C to 24°C, 40%–50% relative humidity, and a 12-hour light/dark cycle. Procedures were performed in accordance with the National Institute of Health's Guide for the Use and Care of Laboratory Animals and were approved by the Committee on Animal Care and Use of the Dongzhimen Hospital. SD rats weighing 380–450 g (*n* = 80) were chosen for a carotid artery model after balloon injury.

### 2.2. Animal Model of Common Carotid Artery Injury

A rat model of the common carotid artery after balloon injury was established to evaluate the vascular remodeling. Sodium pentobarbital 1% (40 mg/kg) was intraperitoneally injected to anaesthetize the rats. The left common carotid artery was isolated through a midline cervical incision to expose a 3 cm segment of the artery from the bifurcation and a 2F Fogarty balloon catheter (diameter of balloon 2 mm and length 20 mm, Baxter Company) was introduced through the left external carotid artery and advanced 4 cm towards the thoracic aorta with the left internal carotid artery blocked. The balloon was inflated with isotonic Na chloride (NS) at 0.5 atm to 0.6 atm to distend the artery and was then pulled back to the bifurcation with constant rotation. This procedure was repeated three times to ensure endothelial denudation and consistent vascular injury. After removing the catheter, the external carotid artery was ligated, the blood flow in the internal carotid artery was restored, the wound closed, and animals were allowed to recover. The external carotid artery of sham operated animal was ligated and common carotid artery was not exposed to balloon injury. Rats were sacrificed and both carotid arteries were collected at 7 or 28 days after balloon injury.

### 2.3. Medications and Grouping

Yiqihuoxuejiedu formula was composed of ingredients such as* Astragalus membranaceus* (Fisch) Bge. var.* mongholicus* (Bge.) Hsiao.,* Salvia miltiorrhiza* Bge.,* Lonicera japonica* Thunb., and* Paeonia suffruticosa* Andr. The formula was produced by the Chinese Herbal Company of Beijing University of Chinese Medicine (Beijing, China) and the final concentration is 1.456 g crude drug/mL.

After balloon injury, rats were randomly divided into 3 groups of 10 rats each: the model group, Captopril group, and Yiqihuoxuejiedu formula group. The sham operated group served as a control. The dosages of Captopril and Yiqihuoxuejiedu formula, which are based on the clinical daily dosages for adult humans with a dose conversion coefficient, were the following: Yiqihuoxuejiedu formula group: 13.368 g/kg/d (corresponding with 12 times the clinical dosages), Captopril group: 12.857 mg/kg/d (12 times clinical dosages). In addition, Acenterine (17.143 mg/kg/d, 6 times) and Pravastatin sodium (1.714 mg/kg/d, 12 times) were used for basic medicine for the Captopril and Yiqihuoxuejiedu groups. Sham operated and model groups received distilled water, 10 mL/kg/d. Medicine was dissolved in distilled water and rats were administered the medicine using a gastric lavage once daily for 7 or 28 days.

### 2.4. Histological and Immunohistochemistry Staining

At 7 or 28 days after balloon injury, animals were euthanized under terminal anaesthesia by exsanguination and retrograde aortic perfusion with 200 mL saline followed by 250 mL saline containing formalin (2% v/v) and glutaraldehyde (0.2% v/v) to fix the tissues before careful excision of the left common carotid artery to avoid any damage to the adventitial layer. The left common carotid arteries were fixed for 24 hours. The slices were stained with Sirius Red and Masson stains as well as haematoxylin and eosin following routine paraffin sections. The expression of smooth muscle *α*-actin was determined in sections incubated in 0.3% hydrogen peroxide for 15 min before using a primary monoclonal *α*-SMA actin antibody (1 : 300, AlPha Sr-1, SANTA) and secondary goat anti-mouse IgG antibody-HRP multimer (DSGB-BIO Origene) and was visualized with diaminobenzidine substrate.

### 2.5. Histological and Immunohistochemistry Quantitative Measurements

The complete cross sections of the left common carotid artery were photographed with a low power lens following haematoxylin and eosin stains to measure and calculate remodeling index including the area of intima, tunica media, and adventitia, as well as stenosis percentage: [(left lumen area around the internal elastic lamina−left narrow lumen area)/left lumen area around the internal elastic lamina] ∗ 100%.

### 2.6. Data Processing Method

Three typical and discrete fields were photographed with high powered lens after immunohistochemical staining. *α*-SMA expression was determined in the intima, tunica media, and adventitia according to brown endochylema. Under high power lens of polarized light, the average optical density and percentage of type I and type III collagen-positive areas were evaluated in 3 representative areas for each compartment of the vessel wall using azure-blue and Sirius Red stained sections.

### 2.7. Statistical Analysis

Statistical analyses were performed using SPSS 11.0 statistics software. The measurement data were presented as the mean ± standard deviation. One-way analysis of variance (ANOVA) followed by Dunnett's test was used to determine the differences among groups. *P* < 0.05 was considered to be statistically significant.

## 3. Results

### 3.1. The Arterial Wall Area after Balloon Injury

#### 3.1.1. Arterial Wall Area at 7 Days after Injury

**Figure 1:**
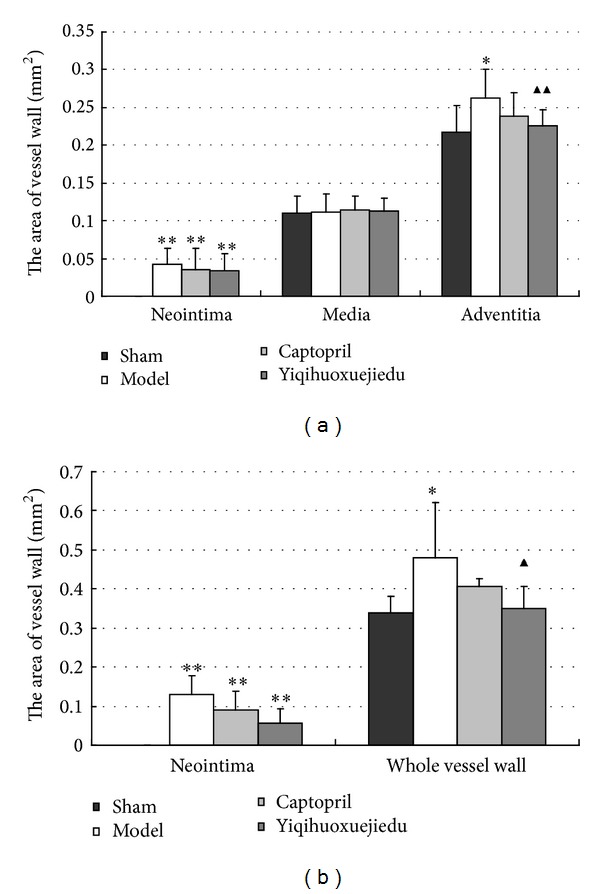
(a) Vessel wall area at 7 days after injury; (b) vessel wall area at 28 days after injury (*P < 0.05 and **P < 0.01 versus the sham group; ^▲^ *P* < 0.05 versus the model group).

**Figure 2:**
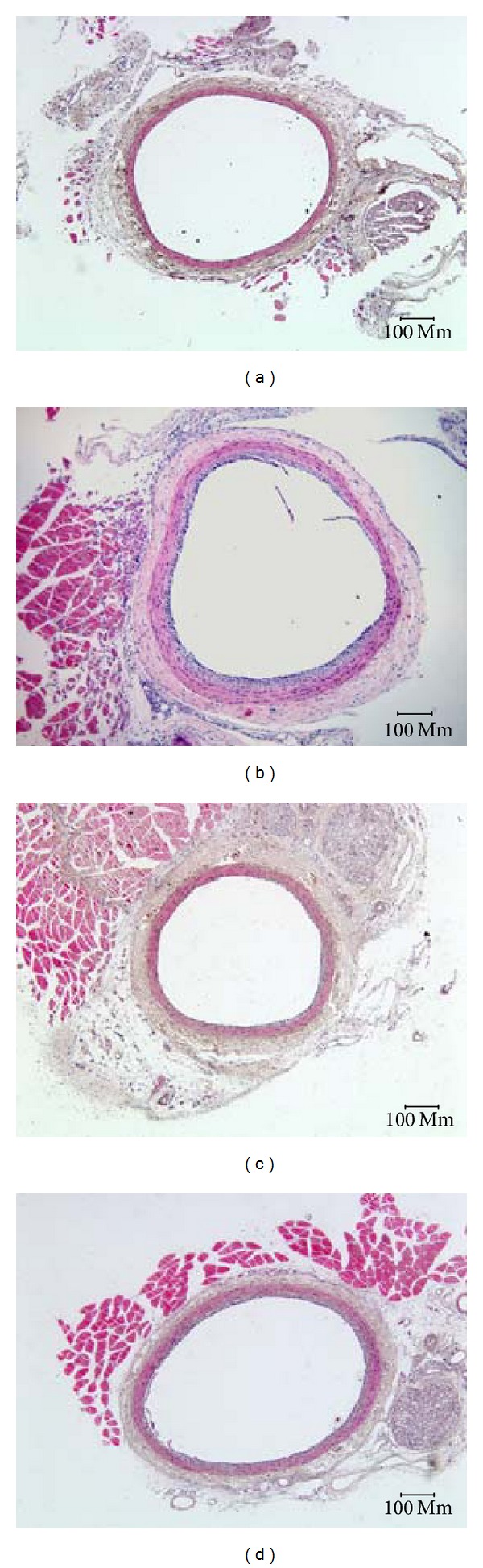
Left common carotid artery slices stained with HE at 7 days after injury. (a) Sham group, (b) model group, (c) Captopril group, and (d) Yiqihuoxuejiedu group.

At 7 days after injury, the areas of neointima and adventitia in the model group and two treatment groups were larger than those of the sham group (*P* < 0.01). There was no change in the medial compartment area among all groups. There was also no significant difference in the neointimal areas of the model and two treatment groups, although the level of two treatment groups decreased to some extent. The Yiqihuoxuejiedu formula reduced the adventitial areas compared with the model group (*P* < 0.01), but Captopril did not (Figures 1(a) and 2).

#### 3.1.2. Arterial Wall Area at 28 Days after Injury

The areas of neointima and of the whole vessel wall were increased in the model group compared with the sham group (*P* < 0.01). The Yiqihuoxuejiedu-treated rats had significantly smaller areas of neointima and the whole vessel wall than did the model rats (*P* < 0.05; Figure 1(b)).

### 3.2. Arterial Stenosis Percentage at 28 Days after Injury

**Figure 3:**
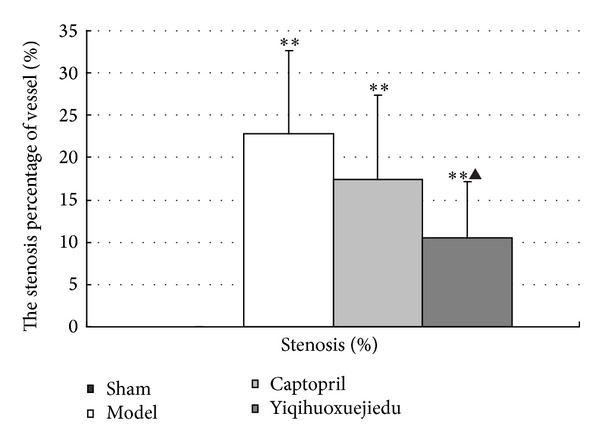
Percentage of arterial stenosis at 28 days after injury (*P < 0.05 and **P < 0.01 versus sham group; ^▲^ *P* < 0.05 versus model group).

At 28 days after injury, the percent stenosis in the model group and two treatment groups increased compared with the sham group (*P* < 0.01 or *P* < 0.05). The Yiqihuoxuejiedu-treated rats had a lower percentage of stenosis occupied by plaque than did the model rats (Figure 3).

### 3.3. The Percentage of Positive Expression of *α*-SMA

#### 3.3.1. The Percentage of Positive Expression of *α*-SMA at 7 Days

**Figure 4:**
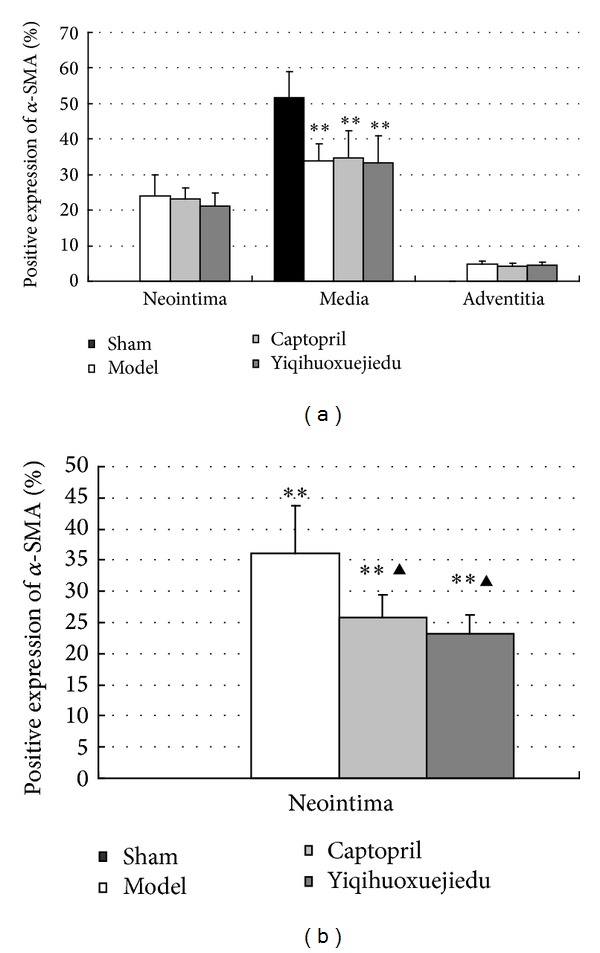
(a) *α*-SMA expression at 7 days after injury; (b) *α*-SMA expression at 28 days after injury (**P < 0.01 versus sham group; ^▲^ *P* < 0.05 versus the model group).

Positive *α*-SMA expression, indicated by brownish yellow or brown granulation in the cytoplasm, was only present in media and was not in the intima and adventitia of the sham group. At 7 days after vascular injury, there was *α*-SMA expression in the media and also in the neointima and adventitia of the model group and the two drug groups, which decreased significantly in the media but increased in the neointima and adventitia compared with the sham group (*P* < 0.01). There was no change in different compartments among the model group and the two drug groups although *α*-SMA expression in the two drug groups decreased (Figure 4(a)).

#### 3.3.2. The Percentage of *α*-SMA Expression at 28 Days

Twenty-eight days after balloon injury, *α*-SMA expression was only seen in the media and there was no expression in the intima and adventitia of the sham group; while there was positive expression in the media and neointima, there was no expression in adventitia of the model and drug groups. The Yiqihuoxuejiedu and Captopril rats had lower *α*-SMA expression in the neointima than the model rats (*P* < 0.01; Figure 4(b)).

### 3.4. The Percent of Collagen at 28 Days after Injury

**Figure 5:**
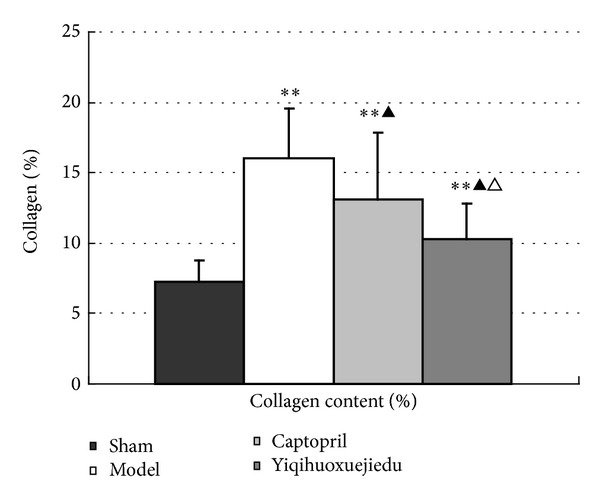
Percent collagen content percent in the vessel wall at 28 days after injury (**P < 0.01 versus the sham group; ^▲^ *P* < 0.05 versus the model group; ^△^ *P* < 0.05 versus the Captopril group).

A large amount of collagen, observed using Masson staining, accumulated in the vessel wall of the model group compared with the sham group (*P* < 0.01). Collagen content of the two drug groups diminished significantly compared with the model group (*P* < 0.01). The Yiqihuoxuejiedu formula reduced collagen hyperplasia more than in the Captopril group (*P* < 0.01; Figure 5).

### 3.5. Ratio of Type I/Type III Collagen in the Adventitia at 28 Days after Injury

**Figure 6:**
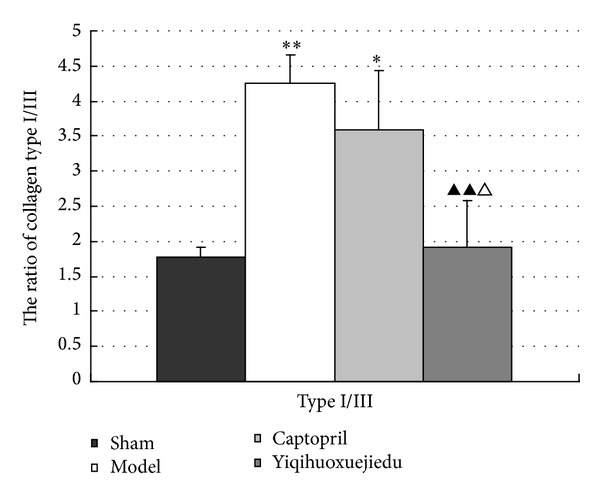
Ratio of type I/type III collagen in the adventitia at 28 days after injury. (*P < 0.05, **P < 0.01 versus sham group; ^▲^ *P* < 0.05 versus model group; ^△^ *P* < 0.05 versus Captopril group).

**Figure 7:**
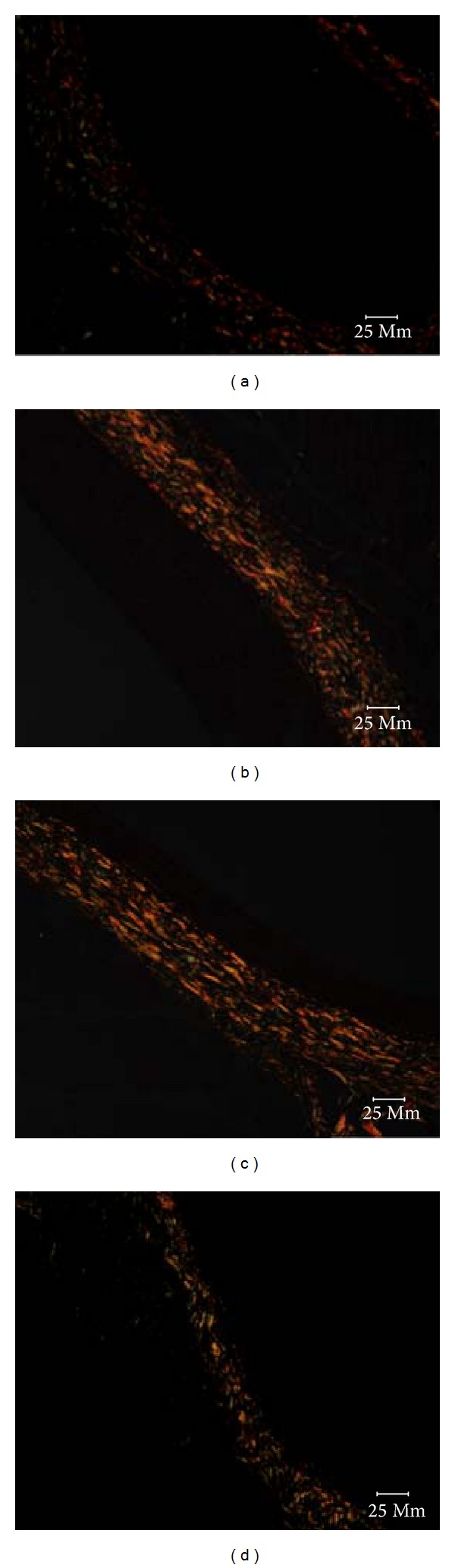
Representative images of adventitia at 28 days after injury (Sirius Red staining ×400). Type I collagen closely spaced and showed strong double refraction, yellow or red fibers. Type III collagen showed weak double refraction, greenish fine fibers. (a) Sham group, (b) model group, (c) Captopril group, and (d) Yiqihuoxuejiedu group.

Rats of the model group obviously increased in the ratio of type I/type III collagen in the adventitia compared with rats in the sham group (*P* < 0.01). There was significant degradation in type I/III collagen in the adventitia of rats in the Yiqihuoxuejiedu group, but there was no difference in the Captopril group compared with the model group (*P* < 0.01). The ratio of types I/III collagen in the Yiqihuoxuejiedu group was lower than that of the Captopril group (*P* < 0.01; Figures 6 and 7).

## 4. Discussion

There are three types of pathological changes in vascular remodeling: vascular structure, cell biology, and function. Structural remodeling refers to transformation of vessel lumen and thickness or area of the vessel wall. Cell biology changes mean that cells in the vascular wall activate, proliferate, migrate, and secrete extracellular matrix (ECM), and the function variation refers to reduced vascular compliance and the reaction to vasoactive substances.

Vascular remodeling is also divided into positive remodeling and negative remodeling. The positive remodeling, also known as compensatory or expansion remodeling, has a compensatory vessel enlargement and relatively sustained changes to the lumen to maintain a constant flow despite an increased plaque burden [12]. Negative remodeling, also called decompensated or constrictive remodeling, causes a reduction in the vessel lumen size that contributes to main pathogenesis of atherosclerosis and restenosis after PCI [12]. The adventitia remodeling can result in either positive or negative remodeling or both. Evidence has specifically shown that the injured adventitia elicits intimal and medial pathological changes that are involved in the evolution of restructuring [4]. Similarly, endothelial damage can lead to pathological processes in the media and adventitia.

Various pathological stimuli (e.g., stretch injury from a balloon angioplasty or endothelial lesion) can induce adventitial fibroblasts differentiation into myofibroblasts (MF, a key player in wound healing and a slow, irreversible retraction [13]) in the adventitia. Differentiated MFs acquire an *α*-smooth muscle actin (*α*-SMA, which is not expressed in normal fibroblasts) phenotype, propagate, and migrate to the neointima, eventually becoming a part of the newly generated intima [14]. These MFs produce a thickened and rigid adventitia rich in collagen fibers leading to adventitia remodeling, consisting of both structural and functional reorganization [15]. In *α*-SMA expression, medial smooth muscle cells (SMC) are opposite to adventitial fibroblasts. SMC in the media has positive *α*-SMA expression in normal conditions, but it loses the *α*-SMA expression following activation of SMC. Therefore, the changes to the *α*-SMA expression in the vascular walls are one of the most important signs of activation in fibroblasts and smooth muscle cells. Vascular remodeling, especially adventitia remodeling, plays an important role in atherosclerosis, hypertension, and restenosis after PCI, and methods of improving vascular remodeling have been recently studied. Chinese medicine is a main part of complementary and alternative medicine and has gained better and more characteristic clinical effects [16]. Xiongshao capsules, which activate the blood circulation, are known to be effective in prevention and treatment of vascular remodeling and restenosis [17, 18]. Yiqihuoxuejiedu formula uses the therapeutic principles of supplementing Qi, activating blood circulation and detoxification based on the Qi blood-related theory of Chinese medicine, and it has effects on inhibiting vascular hyperplasia, reducing blood lipids, and attenuating collagen content in neointima [6, 7]. The formula contains a number of bioactive components. Of these components,* Astragalus* polysaccharide has various important bioactivities, such as immunomodulation, antioxidant, antitumor, antidiabetes, antiviral, hepatoprotection, anti-inflammation, antiatherosclerosis, hematopoiesis, and neuroprotection [19].* Astragalus* membranaceus and* Astragalus *saponin can potently protect endothelium-dependent relaxation against the acute injury from homocysteic acid (HCA) through nitric oxide regulatory pathways [20]. Tanshinone IIA prevents cardiac fibroblast proliferation by interfering with the generation of reactive oxygen species (ROS) and involves the activation of the endothelial nitric oxide synthase-nitric oxide (eNOS-NO) pathway [21]. Tanshinone IIA can also inhibit H_2_O_2_ -induced collagen synthesis via attenuation of O_2_^−^ -generation and nicotinamide adenine dinucleotide phosphate (NADPH) oxidase activity [22]. Paeonol suppresses vascular smooth muscle cell proliferation in vivo and in vitro, which might be related to a decrease in lipid content, lipid peroxidation, and proinflammatory cytokine concentrations. Paeonol also regulates the proliferation periods of vascular smooth muscle cells through inhibiting the PCNA protein expression [23]. Chlorogenic acid has various pharmacologic actions, such as antioxidation, antibacterial, antiviral, antitumor, anti-inflammation, liver protective effect, and antiendotoxin [24]. Salviol IIA and paeonol inhibit adventitial fibroblast hyperplasia and collagen synthesis induced by ANG-II [25]. The above studies suggest that the components in Yiqihuoxuejiedu formula have many kinds of effects, especially on inhibiting fibrosis and reducing cells proliferation and collagen content of vessel wall.

The vascular remodeling mechanism of the formula still requires more research. The present study focuses on the proliferation, activation, and secretion of adventitial fibroblasts following balloon injury and aims to provide experimental evidence on the adventitia. A rat model was established by injuring the common carotid artery, and rats were treated with Yiqihuoxuejiedu formula for 7 or 28 days. At 7 days after injury, the area and *α*-SMA-positive expression in the adventitia significantly increased compared with the sham group, which indicates that adventitial fibroblasts generated clear activation and proliferation. In the sham group, *α*-SMA is expressed in the media rather than in the intima or adventitia. However, in the model group, *α*-SMA has low expression in the media and high expression in the neointima and adventitia. Thus, both adventitial fibroblasts and medial smooth muscle cells are activated, and the former proliferated more than the latter, resulting in larger adventitial areas. At 28 days after injury, some indexes, that is, the area of neointima, percentage of the stenosis, the percentage of collagen content in the vessel wall, and the ratio of type I/type III collagen in the adventitia in the model group, were greater than those of the sham group. These showed that the intimal hyperplasia, vascular stenosis, and vascular stiffness increased, especially in the rigid adventitia that is rich in collagen fibers. Compared with the model group, Yiqihuoxuejiedu formula greatly reduced the adventitial areas but could not suppress *α*-SMA expression in the vessel walls at 7 days after balloon injury. With longer treatment, the formula further reduced neointima and vessel wall areas, diminished the percentage of stenosis, reduced *α*-SMA expression in the neointima, and lowered the collagen content and ratio of type I/type III collagen in the adventitia compared with those of the model group at 28 days. The Yiqihuoxuejiedu formula had better effects than did the Captopril. During the initial period (7 days), Yiqihuoxuejiedu formula inhibited positive (expansionary) remodeling by preventing adventitial hyperplasia, and, in the late stages (28 days), it improved negative (constrictive) remodeling by suppressing neointimal proliferation, reducing the collagen content of the vessel wall and elevating adventitial compliance. However, Yiqihuoxuejiedu formula did not inhibit activation of adventitial fibroblasts (positive *α*-SMA expression in the adventitia) at 7 days. Previous studies of formulas were mainly concerned about intimal thickening and collagen content in vascular wall [26]. Active Components from Chinese herbs had an effect on the proliferation and secretion of smooth muscle cells or adventitial fibroblasts culture in vitro [25]. This study focused on the adventitial remodeling in vivo, especially on adventitial proliferation and collagen changes after intimal damage, and these pathological processes were inhibited by Yiqihuoxuejiedu formula developed from the idea of the surgical wound repair.

In this study, Captopril did not reduce neointimal proliferation or diminish stenosis, although it reduced *α*-SMA expression in the neointima and lowered the collagen content at 28 days. These results might relate to the type of the animal model and mechanism of the angiotensin converting enzyme inhibitor (ACEI) used in this study. ACEI inhibits Ang I conversion to Ang II, which has effects such as constricting blood vessels, increasing blood pressure, and stimulating vascular cell wall proliferation. The level of renin and angiotensin in an injured artery model is not as high as the level in long-term hypertension, which has sustained high levels of Ang II. In addition, ACEI does not completely block effects of Ang II and Ang II still plays a role. This may explain why the effect of ACEI is not significant in this study.

Thus, Yiqihuoxuejiedu formula inhibits positive and negative remodeling by reducing hyperplasia in the adventitia in the early stages and suppresses intimal proliferation, reduces the vessel wall content, and elevates adventitial compliance in the later stages. Inhibiting proliferation and secretion of adventitial fibroblasts is characteristic of the formula. This study suggests new information and a new, complementary method for alternative treatment in the prevention of vascular remodeling, which contributes to further improving atherosclerosis, hypertension, and restenosis after PCI.
